# Hypoxia-induced ZEB1 promotes cervical cancer progression via CCL8-dependent tumour-associated macrophage recruitment

**DOI:** 10.1038/s41419-019-1748-1

**Published:** 2019-07-01

**Authors:** Xiao-Jing Chen, Yuan-Run Deng, Zi-Ci Wang, Wen-Fei Wei, Chen-Fei Zhou, Yan-Mei Zhang, Rui-Ming Yan, Luo-Jiao Liang, Mei Zhong, Li Liang, Sha Wu, Wei Wang

**Affiliations:** 1 Department of Obstetrics and Gynecology, Nanfang Hospital, Southern Medical University, 510515 Guangzhou, China; 2 Department of Obstetrics and Gynecology, The First Affiliated Hospital of Guangzhou Medical University, 510120 Guangzhou, China; 3 Department of Obstetrics and Gynecology, The Third Affiliated Hospital, Southern Medical University, 510360 Guangzhou, China; 4 Department of Immunology, School of Basic Medical Sciences, Southern Medical University, Guangdong Provincial Key Laboratory of Proteomic, 510515 Guangzhou, China; 5 Department of Pathology, Nanfang Hospital, Southern Medical University, 510515 Guangzhou, China; 6 Present Address: 1838 Guangzhou Avenue North, Baiyun District, 510515 Guangzhou, China; 7 Present Address: 151 Yanjiang Road, Yuexiu District, 510120 Guangzhou, China

**Keywords:** Cancer microenvironment, Cervical cancer, Checkpoint signalling

## Abstract

The accumulation of tumour-associated macrophages (TAMs) in the hypoxic tumour microenvironment (TME) is associated with malignant progression in cancer. However, the mechanisms by which the hypoxic TME facilitates TAM infiltration are not fully understood. This study showed that high ZEB1 expression in hypoxic cervical cancer cell islets was positively correlated with CD163^+^ TAM accumulation. ZEB1 in hypoxic cancer cells promoted the migration of TAMs in vitro and altered the expression of multiple chemokines, especially CCL8. Mechanistically, hypoxia-induced ZEB1 activated the transcription of CCL8, which attracted macrophages via the CCR2–NF-κB pathway. Furthermore, ZEB1 and CCL8 were independent prognostic factors in cervical cancer patients based on The Cancer Genome Atlas (TCGA) data analysis. In conclusion, hypoxia-induced ZEB1 exerts unexpected functions in cancer progression by fostering a prometastatic environment through increased CCL8 secretion and TAM recruitment; thus, ZEB1 may serve as a candidate biomarker of tumour progression and provide a potential target for disrupting hypoxia-mediated TME remodelling.

## Introduction

Tumour hypoxia is an adverse factor in cervical cancer, and it is associated with a poor outcome regardless of the treatment modality^1^. It is well established that a hypoxic tumour microenvironment (TME) promotes tumour malignant progression and limits the effectiveness of solid tumour immunotherapies by promoting neoplastic transformation and inducing tumour cell resistance to host immunity^2^. The manipulation of hypoxic stress in future cancer immunotherapy approaches is of major interest.

Increasing evidence has demonstrated that tumour hypoxia affects the antitumour immune response by promoting local immune suppression and inhibiting immune killing functions^3^. Hypoxic zones in solid tumours are infiltrated by a large number of immunosuppressive cells, such as tumour-associated macrophages (TAMs), myeloid-derived suppressor cells (MDSCs) and T-regulatory (Treg) cells. These cells are among the most widely studied immunosuppressive cells within the TME, and the role of tumour hypoxia in their recruitment and immunosuppressive functions has become clear^4,5^. Macrophages constitute a principal component of the immune infiltrate in solid tumours, and their heterogeneity in different microenvironments results in different immune responses^6^. In our previous study, macrophages in tumour hypoxic niches differentiated into TAMs with an immunosuppressive phenotype^7^, and the presence of high numbers of these cells has been strongly associated with poor patient outcome^8^. However, how these hypoxia-primed cancer cells facilitate TAM infiltration through crucial tumour–host interactions during cancer progression is unclear.

Chemokines are necessary for macrophage migration. Previous studies reported that CCL2 and CSF-1 are the main chemokines required for macrophage migration into the TME^9–11^. However, there is a different immunophenotype in normoxic and hypoxic TMEs, including the cytokine-releasing secretome. Epithelial–mesenchymal transition (EMT) is frequently observed at the hypoxic invasive front of tumours^12^, which is where TAMs are usually located^8^. The relationship between cancer cell EMT and macrophage chemokines in the hypoxic TME may explain TAM accumulation in the hypoxic tumour zone. A key regulator of EMT in cancer cells at the invasive front is the transcription factor ZEB1, which endows cancer cells with a pro-invasive and mesenchymal-like phenotype and predicts a worse clinical prognosis in most cancers^13^. Since TAMs, which are preferentially located in the hypoxic invasive front that undergoes EMT, are critical players in the interactions between cancer cells and their microenvironment, we sought to investigate whether ZEB1 plays a role in the crosstalk between cancer cells and TAMs in the hypoxic TME.

TAMs in the hypoxic milieu constitute an important target in cancer therapy, and efforts are being made to regulate their recruitment and pro-tumour functions^8,14^. Our study revealed a possible mechanism of TAM infiltration into the hypoxic tumour zone via ZEB1-driven cervical cancer cells. The unexpected role of hypoxia-induced ZEB1 established by our study offers additional approaches to target TAMs in cancer therapy.

## Materials and methods

### Cell lines

Human cervical squamous carcinoma cell lines (SiHa and C33a) and murine RAW264.7 macrophages were purchased from ATCC and cultured according to their guidelines_._ Human monocytic cell line THP-1 was obtained from the China Center for Type Culture Collection (CCTCC) and cultured in RPMI 1640 medium supplemented with 10% fetal bovine serum. Hypoxia treatment was administered by placing the cells in a hypoxic incubator (Thermo Scientific, USA) maintained at a low oxygen concentration (1% O_2_, 5% CO_2_ and 94% N_2_).

### Clinical specimens

A total of 90 archived, formalin-fixed and paraffin-embedded cervical cancer specimens were obtained from the Department of Gynecological Oncology of Nanfang Hospital (Guangzhou, China) between 2013 and 2014. The study was approved by the Institutional Research Ethics Committee of Southern Medical University. Details of the clinicopathologic characteristics are provided in Table 1. None of the patients had received preoperative chemotherapy or radiotherapy. Each section was evaluated by two experienced pathologists who were blinded to the patients’ clinicopathological data.

**Table 1:** Association of the clinicopathologic variables with the expression levels of CAIX, ZEB1 and CD163

Clinicopathological indexes	No. of patients	CAIX			ZEB1			CD163		
		Low	High	*P*	Low	High	*P*	Low	High	*P*
Age (years)				0.162			0.458			0.143
<45	42	21	21		26	16		18	24	
≥45	48	31	17		26	22		28	20	
FIGO stage				0.036^a^			0.022^a^			0.012^a^
I–IIA	67	43	24		47	20		46	21	
IIB–IV	23	9	14		10	13		9	14	
Histology				0.369			0.385			0.832
High	22	12	10		9	13		11	11	
Middle	46	33	13		27	19		25	21	
Low	22	14	8		12	10		13	9	
LN metastasis				0.030^a^			0.018^a^			0.019^a^
Negative	50	35	15		30	20		26	24	
Positive	40	19	21		14	26		11	29	

*LN* lymph node

^a^Statistical significance *(P* < 0.05) was calculated using the Chi-squared test

### Western blot assay

Western blot assays were performed as previously described^7^. The primary antibodies were as follows: anti-CAIX (#ab184006; Abcam), anti-ZEB1 (#DF7414; Affinity), anti-NF-κB (#8242; CST), anti-p-NF-κB (#3033; CST), anti-AKT (#4685; CST), anti-p-AKT (#4060; CST), anti-ERK1/2 (#4695; CST), anti-P-ERK1/2 (#4370; CST), anti-p38 (#8690; CST) and anti-p-p38 (#4511; CST).

### Quantitative reverse transcription-polymerase chain reaction

Total RNA was extracted from SiHa and C33a cells by Trizol Reagent (Solarbio, China). Quantitative reverse transcription-polymerase chain reaction (qRT-PCR) was performed as previously described^7^. The primer set for CCL8 was purchased from Invitrogen (sense: 5′-TGGAGAGCTACACAAGAATCACC-3′, antisense: 5′-TGGTCC AGATGCTTCATGGAA-3′). The mRNA expression was normalized to GAPDH (sense: 5′-CCATCAATGACCCCTTCATTGACC-3′, antisense: 5′-GAAGGCCATGCCAGT GAGCTT CC-3′).

### Immunofluorescence

Serial paraffin sections (4 μm) from human cervical cancer tissues were analysed by immunofluorescence with an Opal 4-Color Kit (PerkinElmer) according to the manufacturer’s protocol^15^. Briefly, sections were microwaved in antigen retrieval buffer for 15 min at 90 °C after deparaffinization; then, they were washed and blocked for 10 min at room temperature and incubated with an anti-ZEB1 antibody. Horseradish peroxidase-conjugated secondary antibody was dropped onto slides for incubation for 10 min at room temperature. Subsequently, tyramide signal amplification (TSA) buffer (Opal 650) was used to amplify the signal on the slides. After eliminating the anti-ZEB1 and secondary antibodies by microwaving, the above procedures were repeated with an anti-CAIX antibody and TSA buffer (Opal 570). The above procedures were repeated again with an anti-CD163 antibody (ab156769; Abcam) and TSA buffer (Opal 520). The sections were mounted in neutral gum and visualized by a fluorescence microscope (Olympus).

### Cell transfection

Cells were transfected with 10 µM ZEB1 siRNA or 4 µg ZEB1 expression plasmid (GenePharma, China) using Lipofectamine™ 2000 (Invitrogen, USA) according to the manufacturer’s instructions. The transfection efficiency was greater than 90%, as confirmed by the detection of FITC-labelled siRNA (Invitrogen, USA).

### Transwell migration assay

THP-1 monocytes were primed with 5 nM PMA (Sigma) for 24 h to become monocyte-derived macrophages as previously described^16^. Transwell assays assessing cell migration potential were performed on 24-well plates with inserts (8-μm pore size; Millipore) as previously described^7^. Briefly, 5 × 10^5^ of primed THP-1 cells or RAW264.7 cells were cultured in the upper chamber and allowed to migrate for 24 h before fixation and crystal violet staining. The concentration of CCL8 was selected according to previous literature reports^17^. Cells were counted from five random fields of view. The data are represented as the mean ± SD, and all experiments were repeated at least three times independently.

### Immunohistochemistry

Tissue sections were subjected to immunohistochemistry analysis as previously described^7^. The primary antibodies were as follows: CAIX, ZEB1 and CD163. The secondary antibodies were horseradish peroxidase-conjugated anti-rabbit and mouse immunoglobulin-G antibody (both applied at 1:5000; Abcam).

### Evaluation of immunohistochemistry results

The counting was performed using the *H* score algorithm^18^. Fields at ×400 magnification were used for *H* score assessment, and the staining intensity in the malignant cells was scored as 0, 1, 2, or 3, corresponding to the presence of negative, weak, intermediate, or strong brown staining, respectively. The total number of cells in each field and the number of cells stained at each intensity were counted. The *H* score was calculated as follows: (% of cells stained at intensity 1 × 1) + (% of cells stained at intensity 2 × 2) + (% of cells stained at intensity 3 × 3). An *H* score between 0 and 300 was obtained, where 300 was equal to 100% of the tumour cells stained strongly (3+). The median *H* score values were selected to distinguish between low and high CAIX or ZEB1 expression groups. The density of CD163 + TAMs was counted as previously described^19^.

### Enzyme-linked immunosorbent assay

The CCL8 level in the conditioned media (CM) of cervical cancer cells was measured using an ELISA kit (#442207; Biolegend) according to the manufacturer’s instructions. Briefly, a 96-well microplate was precoated with an anti-human CCL8 antibody. First, 100 µL of each standard or sample was added to the appropriate wells and incubated for 2 h at RT with gentle shaking. After discarding the solution and washing four times, 100 µL of human CCL8 detection antibody was added to each well, and incubated for 1 h. After washing away the unbound biotinylated antibody, 100 µL of horseradish peroxidase (HRP)-conjugated streptavidin was added to the wells and incubated for 30 min, and 100 µL of tetramethylbenzidine one-step substrate reagent was added after five washes. Subsequently, 50 µL of stop solution was added to each well, and the plate was immediately read at 450 nm.

### Dual-luciferase assays

The expression of the ZEB1-targeted genes was measured using a dual-luciferase reporter assay in SiHa and C33a cells as previously described^20^. Luciferase activity was measured 48 h after transfection using the Dual-Luciferase Reporter Assay System. Each assay was repeated in three independent experiments.

### Data source

The gene expression dataset from human cervical cancer patients was obtained from the University of California, Santa Cruz Xena browser (UCSC Xena: http://xena.ucsc.edu/, accessed January 7, 2019). The corresponding clinical information from The Cancer Genome Atlas (TCGA) cervical cancer patients was downloaded from TCGA (https://portal.gdc.cancer.gov/, accessed January 7, 2019). The datasets included in the current study were downloaded from public databases; therefore, there was no need for the study to be approved by an additional ethics committee.

### Statistical analysis

SPSS (version 20.0) software was used for statistical analysis. The results are expressed as the mean value ± SEM and were analysed by *t*-test. Frequency tables were analysed using the Chi-squared test, and Pearsons’ correlation coefficient was used to assess the significance of the correlations between categorical variables. Univariate survival analysis for ZEB1 and CCL8 was performed using the log-rank test. Differences were considered to be statistically significant when *P* < 0.05.

## Results

### Hypoxia-induced ZEB1 is positively associated with TAM distribution and cervical cancer progression in clinical specimens

**Fig. 1:**
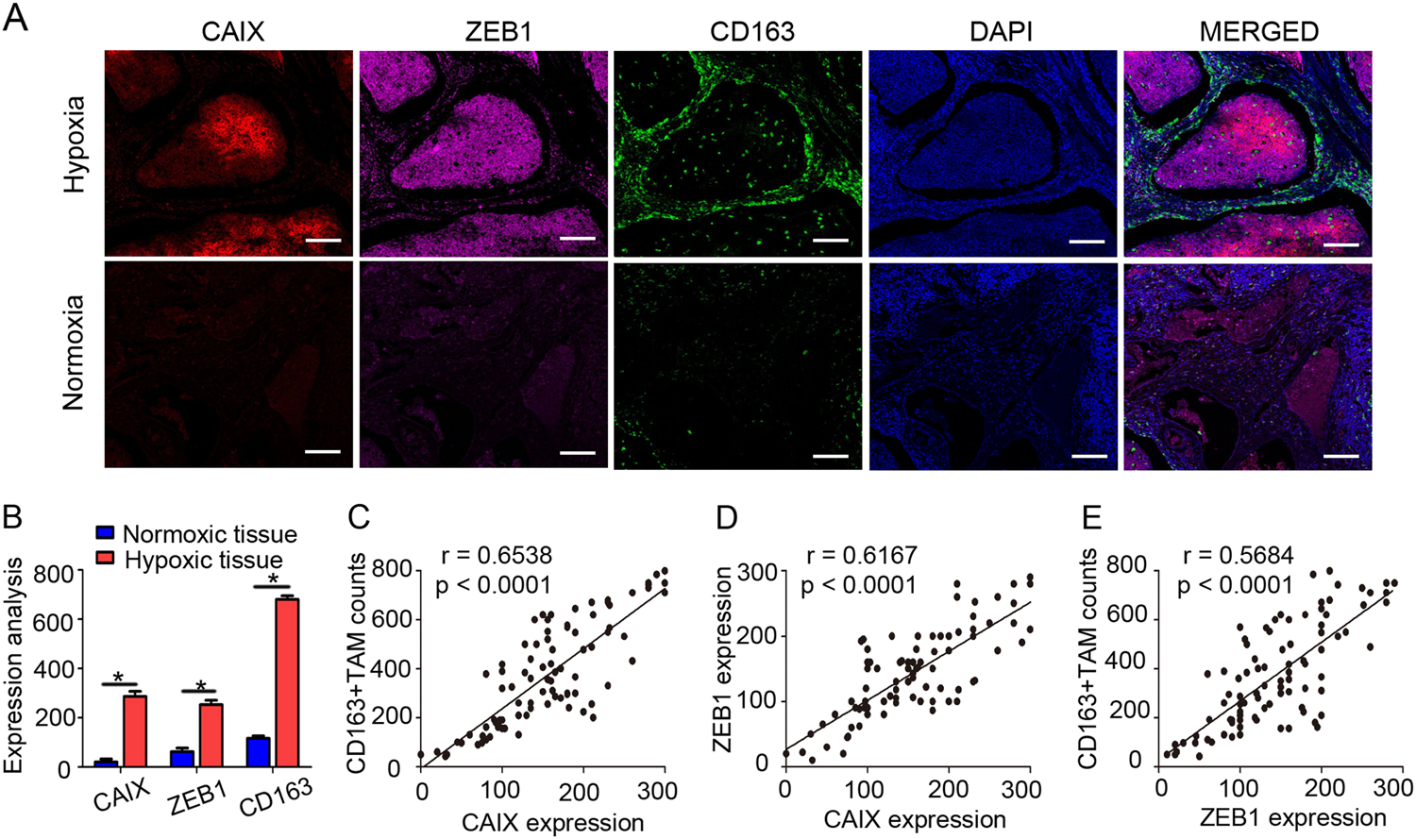
The number of TAMs is correlated with ZEB1 expression in the hypoxic regions of cervical cancer. **a** Immunofluorescence staining of CAIX, ZEB1, and CD163^+^ TAMs in normoxic and hypoxic areas of human cervical cancer tissues (scale bar, 100 μm). **b** Statistical analysis showing that the levels of CAIX, ZEB1, and CD163^+^ TAMs between normoxic and hypoxic tissues were significantly different. *P < 0.05 by Student’s *t*-test. **c**–**e** The clinical relevance of CAIX, ZEB1, and CD163^+^ TAMs was analysed by Pearsons’ coefficient test

Hypoxia is a hallmark of solid tumours and leads to mesenchymal-like morphological changes in cervical cancer cells^21^. Since ZEB1 is best known for driving EMT in cancer cells, we then tested its function in the cervical hypoxic TME and its correlation with TAM infiltration. Immunofluorescence staining was applied to analyse the expression of carbonic anhydrase IX (CAIX, an established cellular biomarker of hypoxia^22,23^), ZEB1 and CD163 (a TAM marker^24^) in tissues derived from human cervical cancer patients. As shown in Fig. 1a, intense ZEB1 staining was observed in the hypoxic regions where CAIX was expressed abundantly, and ZEB1 was mainly distributed within cancer cell islets. In accordance with ZEB1 deposition, considerable CD163^+^ TAMs infiltrated the stroma surrounding the hypoxic cancer cell islets but not the normoxic regions. In contrast, ZEB1 was undetectable in CAIX-deficient normoxic regions, where CD163^+^ TAMs were also hardly detected. Statistical analysis showed that the levels of CAIX, ZEB1 and CD163^+^ TAMs between normoxic and hypoxic tissues were significantly different (Fig. 1b, *P* < 0.05). Clinical relevance was analysed by Pearsons’ coefficient test, and significant correlations between CAIX and CD163^+^ TAMs, CAIX and ZEB1, and ZEB1 and CD163^+^ TAMs were observed (Fig. 1c–e, *r* = 0.6538, *P* < 0.0001; *r* = 0.6167, *P* < 0.0001; *r* = 0.5684, *P* < 0.0001, respectively). Collectively, the results led us to conclude that levels of ZEB1 were upregulated in the hypoxic area of human cervical cancer specimens, which in turn coincided with higher accumulation of CD163^+^ TAMs.

To further understand their clinical value, we examined the relationships among CAIX expression level, ZEB1 expression level, TAM density, and clinicopathologic characteristics. As shown in Table 1, increased CAIX intensity, upregulated ZEB1 expression level, and increased number of CD63^+^ TAMs were strongly correlated with an advanced FIGO stage and lymph node (LN) metastasis of cervical cancer (all *P* < 0.05). These findings support the concept that TAMs in hypoxia contribute to the progression of human cervical cancer.

### Hypoxia-induced ZEB1 in cancer cells enhances TAM migration in vitro

**Fig. 2:**
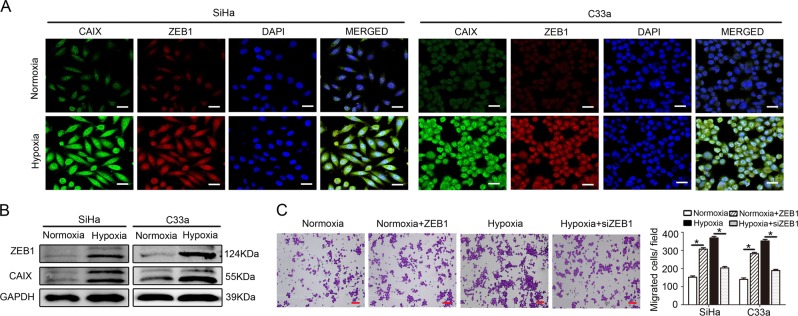
Hypoxia-induced ZEB1 in cancer cells enhances TAM migration in vitro. **a** Immunofluorescence revealed that hypoxic SiHa and C33a cells exhibited upregulated expression of CAIX (green) and ZEB1 (red). Nuclei were stained with DAPI (blue) (scale bar, 50 μm). **b** Western blotting shows that the protein levels of both ZEB1 (upper panel) and CAIX (middle panel) were higher in hypoxic cancer cells than in normoxic cancer cells (left panel shows SiHa cells; right panel shows C33a cells). **c** The effect of conditioned media (CM) from hypoxia-treated cervical cancer cells on THP-1 cells was detected by transwell migration assay. Scale bar, 50 μm. *P < 0.05 by Student’s *t*-test

We analysed the effect of hypoxia on ZEB1 expression in cervical cancer cells (SiHa and C33a) in vitro with immunofluorescence staining and western blotting. SiHa and C33a cells were initially exposed to hypoxic conditions for 48 h as previously described. Cells maintained under normoxic conditions served as controls. The results revealed that the expression levels of CAIX and ZEB1 were both highly increased in SiHa and C33a cells under hypoxic culture conditions (Fig. 2a, b).

We further detected the effect of CM from hypoxia-treated cervical cancer cells on macrophages. As shown in Fig. 2c and Supplementary Fig. S1, CM from hypoxia-primed SiHa and C33a cells recruited more macrophages than CM from normoxic cells (all *P* < 0.05). To further investigate ZEB1 function on macrophage migration, we established ZEB1-overexpressing normoxic cervical cancer cells and ZEB1-silenced hypoxic cervical cancer cells. After silencing ZEB1 in hypoxic cervical cancer cells, the recruitment of macrophages was decreased, and overexpression of ZEB1 in normoxic cervical cancer cells increased macrophage migration (all *P* < 0.05). Furthermore, our previous study verified that a cervical hypoxic TME induced the formation of TAMs with a pro-tumour phenotype^7^. These results suggest that hypoxia-induced ZEB1 in cancer cells enhances TAM migration in vitro.

### CCL8 is the critical chemokine induced by hypoxic cancer cell-derived ZEB1 to attract TAMs

**Fig. 3:**
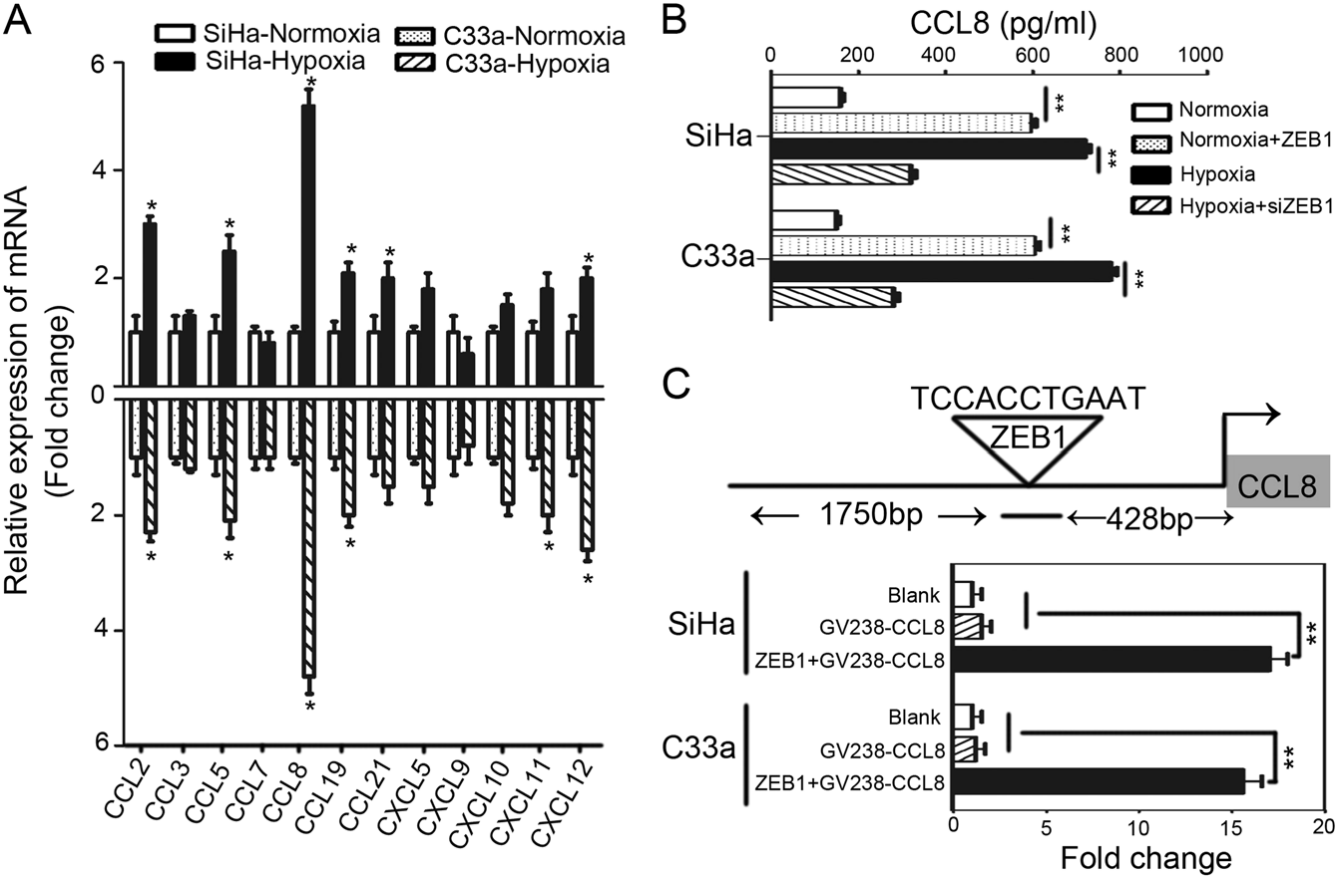
CCL8 is the critical chemokine induced by hypoxic cancer cell-derived ZEB1 to attract TAMs. **a** Multiple related chemokines were screened in normoxic and hypoxic cervical cancer cells by qRT-PCR. **b** Levels of CCL8 secreted by cervical cancer cells were measured by enzyme-linked immunosorbent assay. **c** A dual-luciferase reporter assay system was performed to demonstrate the direct binding of ZEB1 to the CCL8 promoter region. **P < 0.01, *P < 0.05 by Student’s *t*-test

To further identify the possible target genes of ZEB1, candidate chemokines were screened through RT-qPCR analysis. As shown in Fig. 3a, hypoxia promoted cancer cell production of more macrophage chemokines, and among all increased chemokines (CCL2, CCL5, CCL8, CCL19, CCL21, and CXCL1), CCL8 was the most increased according to qRT-PCR detection. Consistently, an increased level of CCL8 in the CM of hypoxic cervical cancer cells was also demonstrated by enzyme-linked immunosorbent assay (Fig. 3b).

In addition, CCL8 upregulation was detected in ZEB1-transduced normoxic cervical cancer cell CM, and CCL8 was decreased in ZEB1-silenced hypoxic cervical cancer cell CM, suggesting that ZEB1 controls CCL8 production in cancer cells (Fig. 3b). To uncover the molecular mechanisms of ZEB1-induced CCL8 secretion, the JASPAR 2018 database was used for analysis. As shown in Fig. 3c, the RNA sequence of the predicted ZEB1-binding site to CCL8 was TCCACCTGAAT, which suggested that ZEB1 was a putative upstream transcription factor of CCL8. We further performed a dual-luciferase reporter assay to demonstrate the direct binding of ZEB1 to the CCL8 promoter region. The promoter sequence of CCL8, which harbours a complementary sequence for the ZEB1 seed sequence, was cloned into a luciferase reporter plasmid. As shown in Fig. 3c, transient cotransfection of the CCL8 promoter construct with ZEB1 into SiHa and C33a cells led to a significant increase in firefly luciferase activity compared to the control conditions (**P < 0.01). The results suggest that ZEB1 could directly target the CCL8 promoter in cervical cancer cells.

### CCL8–CCR2 interaction mediates TAM infiltration in hypoxic conditions

**Fig. 4:**
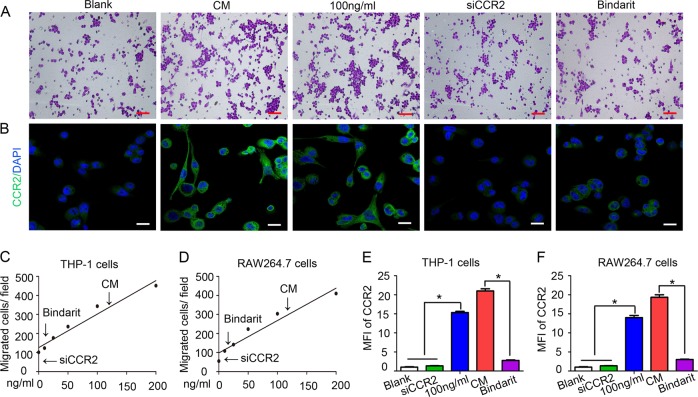
CCL8–CCR2 interaction mediates TAM infiltration under hypoxic conditions. **a** Using a migration test, we identified that the migration effect of hypoxic cervical cancer cells on THP-1 cells was almost equivalent to that of 100 ng/ml CCL8. Bindarit (a CCL8 synthesis inhibitor) and siCCR2 significantly impaired the directional migration of THP-1 cells. Scale bar, 100 μm. **b** Immunofluorescence staining revealed that the level of CCR2 expression was increased in THP-1 cells incubated with 100 ng/ml CCL8 and decreased in THP-1 cells treated with bindarit or siCCR2. Scale bar, 100 μm. **c** Statistical analysis showing the number of migrated THP-1 cells. **d** Statistical analysis showing the number of migrated RAW264.7 cells. **e** Statistical analysis showing the fold change of the mean fluorescence intensity (MFI) of CCR2 in THP-1 cells. **f** Statistical analysis showing the fold change of the MFI of CCR2 in RAW264.7 cells

We performed a transwell migration assay to validate whether CCL8 functions as a pivotal chemoattractant of TAMs. Using a migration test, we identified that the migration effect induced by hypoxic cervical cancer cells on macrophages was almost equivalent to that of 100 ng/ml CCL8 (Fig. 4a shows THP-1 cells; Supplementary Fig. S2A shows RAW264.7 cells; Fig. 4c, d). Consistently, the migration-promoting effect of hypoxic cervical cancer cells on macrophages was inhibited by a CCL8 inhibitor (bindarit). Moreover, blockade of the CCL8 receptor CCR2 (siCCR2) in macrophages significantly impaired macrophage migration (all *P* < 0.05). As shown in Fig. 4b and Supplementary Fig. S2B, immunofluorescence staining revealed that the expression of CCR2 in macrophages increased with CM and CCL8 stimulation and decreased with CCL8 inhibition and CCR2 silencing. The statistical analyses in Fig. 4e, f show the fold change of the mean fluorescence intensity in macrophages. These data demonstrate that the CCL8–CCR2 axis plays a critical role in the recruitment of TAMs by hypoxic cervical cancer cells.

### CCL8 regulates NF-κB signalling to recruit TAMs

**Fig. 5:**
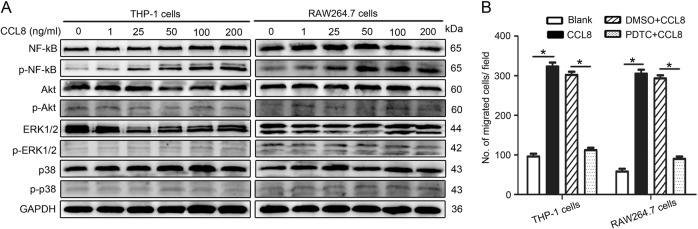
CCL8 regulates NF-κB signalling to recruit TAMs. **a** We searched the related downstream signals for CCL8 by western blot in both macrophage cell lines. **b** Obvious inhibition of the recruitment of THP-1 cells and RAW264.7 cells was found in the cells incubated with PDTC (an inhibitor of NF-kB signalling). Scale bar, 100 μm. *P < 0.05 by Student’s *t*-test

To further elucidate the possible pathway that may be involved in the recruitment of TAMs by CCL8, we investigated the migration-related NF-κB, AKT, ERK1/2, and p38 signalling pathways. The results showed that NF-κB phosphorylation (p65) in THP-1 cells was considerably increased after exposure to CCL8, similar to that in RAW264.7 cells (Fig. 5a). To further identify the function of NF-κB signalling in mediating the recruitment of TAMs by cancer cells, a specific inhibitor of NF-κB signalling, PDTC (pyrrolidine dithiocarbamate ammonium), was used to inhibit the translocation and accumulation of NF-κB in the nucleus^25^. After inhibition of NF-κB, CCL8 attraction of macrophages was obviously halted (Fig. 5b), supporting the critical role of the NF-κB signalling pathway in CCL8 recruitment of macrophages.

### ZEB1 and CCL8 are corrected with poor prognosis in cervical cancer patients

**Fig. 6:**
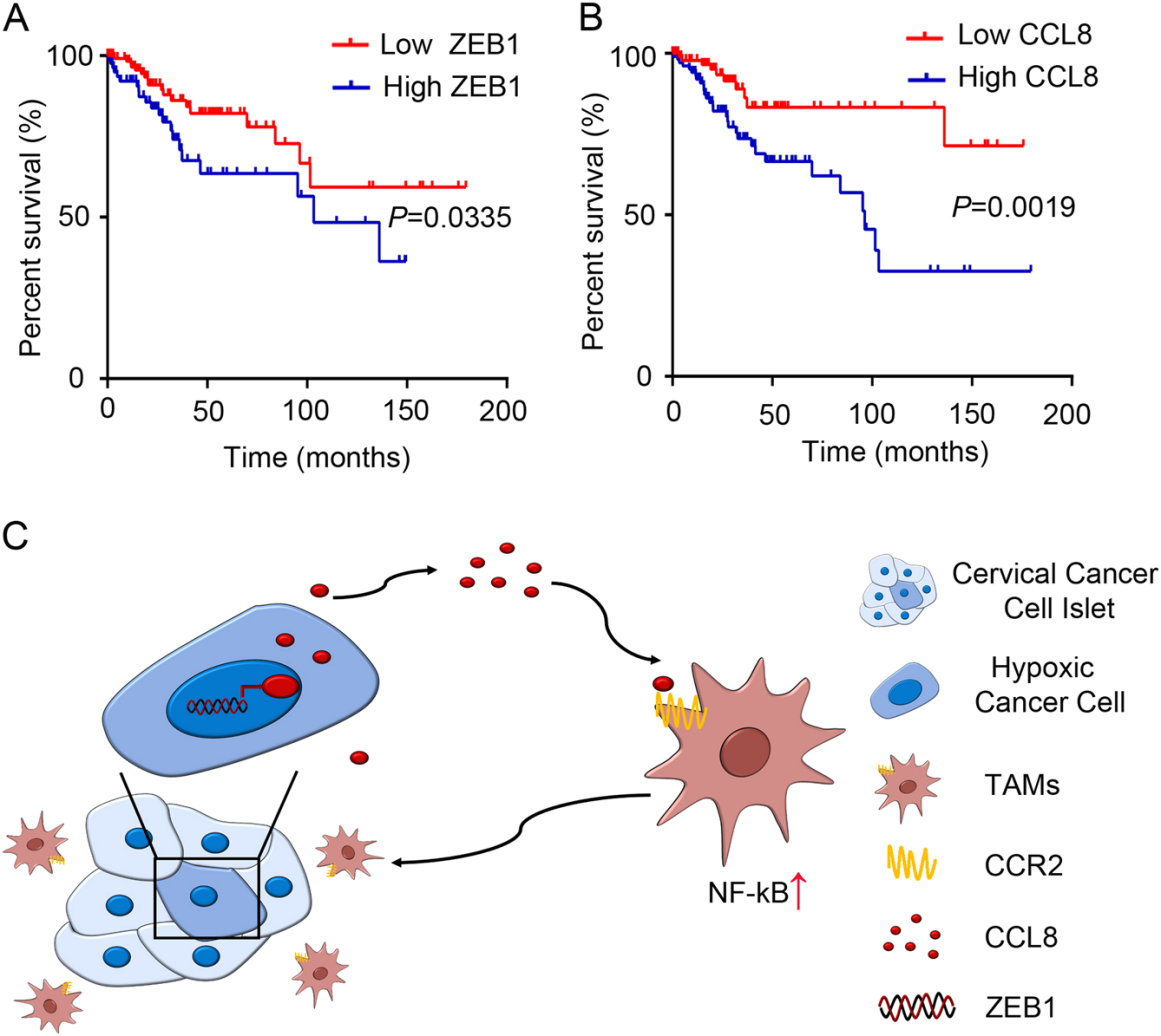
ZEB1 and CCL8 are independent prognostic factors in patients with cervical cancer. TCGA data showed that ZEB1 (**a**) and CCL8 (**b**) overexpression correlated with poor prognosis in human cervical cancer (*P* = 0.0335, *P* = 0.0019, respectively). *P < 0.05 by log-rank test. **c** Illustrative model showing the proposed mechanism by which hypoxia-induced ZEB1 potentiates TAM infiltration through its promotion of the secretion of CCL8 to form a metastasis-favourable TME

To evaluate the relevance of our observations to clinical prognosis, public data from TCGA were analysed. TCGA data analysis showed that the increased expression levels of ZEB1 and CCL8 were correlated with poor prognosis in human cervical cancer (Fig. 6a, b, *P* = 0.0335, *P* = 0.0019, respectively), which further suggested that ZEB1 and CCL8 may play oncogenic roles in the progression and development of cervical cancer.

Collectively, we propose a model to summarize our observations in this study in Fig. 6. Hypoxia increased ZEB1 expression in cervical cancer cells, directly promoting CCL8 production and attracting macrophages via the CCR2–NF-κB pathway. CCL8-mediated TAM infiltration contributes to hypoxic ZEB1-related cancer progression.

## Discussion

CCL2 and CSF-1 are thought to be critical chemokines that attract macrophages to the TME^9,11,26^. However, compared to the normoxic TME, the hypoxic TME was recently found to have a more immunosuppressive profile, including TAM accumulation, MDSC formation, T cell exhaustion and metabolism, and cytokine release^3,27^. Our study first uncovered that in the hypoxic TME, CCL8 from hypoxia-induced ZEB1-driven cancer cells induced macrophage infiltration mainly into the hypoxic area via the CCR2–NF-κB pathway, and this effect was associated with poor prognosis in cervical cancer.

Hypoxia represents a key micro-environmental stressor that governs multiple stronger immunosuppressive phenomena associated with tumour progression^28^. Hypoxia promotes the infiltration of MDSCs and Tregs and stimulates the expression of the immune checkpoint molecules PD-L1 and IDO in tumour cells and stromal cells, which collectively exhaust cytotoxic T cells^29^. TAMs preferentially accumulate in the hypoxic areas of tumours;^14^ this process plays a prominent role in tumourigenesis and could release factors that promote tumour growth and metastatic dissemination^30^, but the exact underlying mechanism remains elusive. In this study, we observed an interesting phenomenon in which TAMs in the stroma always surrounded ZEB1-positive cells, supporting the unexpected role of ZEB1 in the recruitment of TAMs. We also found that ZEB1 was highly expressed in hypoxic cervical cancer cell islets, which are known to display a stronger pro-tumour phenotype. Hypoxia induces ZEB1 expression by the direct binding of HIF-1α to the proximal promoter of ZEB1 via hypoxia response element sites, thus increasing the transactivity and expression of ZEB1 (ref. ^31^). Moreover, the higher expression of ZEB1 in cervical cancer is positively correlated with advanced FIGO stage and LN metastasis. Previous studies demonstrated the pro-tumour effect of ZEB1 by revealing its stimulatory effect on EMT^32^. Inhibition of ZEB1 abrogated HIF-1α-induced EMT and cell invasion^33^. Our study discovered its novel function in mediating the signalling between hypoxia and TAMs. Interestingly, ZEB1 is also induced in TAMs to maintain the pro-tumour phenotype^34^. These results suggest that hypoxia-induced ZEB1 in cancer cells promotes tumour metastasis in a TAM-dependent manner.

The different chemokine patterns expressed in different tumour zones play a vital role in the orientation and differentiation of macrophages, which modulate the suitability of the TME for cancer progression^35^. Previous studies suggest that tumours may recruit TAMs to the TME via the secretion of CCL2 and CSF-1 (refs. ^9,11^). However, we found that the hypoxic tumour area displayed a different chemokine release pattern than the normoxic tumour area. Our study identified that in the hypoxic zone, cancer-derived CCL8 is the most upregulated chemokine, and it attracts macrophages to the hypoxic TME, which is related to the malignant progression of cervical cancer. This study revealed that hypoxia-governed CCL8, which is more important than CCL2 and CSF-1, induced TAM migration. Mechanistically, we also elucidated that hypoxia-induced ZEB1 activated the transcription of CCL8. A poor prognosis was found in cervical cancer patients with high expression of ZEB1 and CCL8. As a known potent macrophage attractor, CCL8 is abnormally increased in various types of tumours and functionally contributes to tumour metastasis; it has also been determined to be an effective marker to predict the prognosis of tumour patients^36,37^. In addition, CCL8 contributes to the migration property and EMT process of cancer cells^16,38^. Therefore, exploring the biological characteristics and molecular mechanisms underlying sustained CCL8 expression in cervical cancer may provide clinically predictive tools for finding effective anti-CCL8 treatments.

Recent evidence indicates that hypoxia can strictly control the chemokine network not only by regulating the production of specific chemokines but also by controlling their action through the modulation of their receptors^14,39^. In this study, we found that the level of CCR2 expression increased in macrophages incubated with CCL8, and the blockade of CCR2 almost halted TAM migration, suggesting that the recruitment of macrophages by CCL8 is mediated mainly by CCR2. Moreover, CCR2 activation in macrophages is essential to their polarization towards a pro-tumour phenotype^34^. CCR2 was reported to be the key receptor for CCL8 in macrophages^40,41^. In addition to CCR2, CCL8 elicits chemoattraction by binding to other membrane receptors, including CCR1, CCR3, CCR5, and CCR8 (ref. ^42^). However, the expression of these receptors in macrophages was significantly decreased under hypoxic conditions^14,43^. Although CCL2 is also a ligand of CCR2, we found that CCL8 expression was increased more than CCL2 expression in hypoxic cervical cancer cells, competitively binding with CCR2, which mediated macrophage infiltration^40^. Consistently, the migration-promoting effect of hypoxic cervical cancer cells on macrophages was inhibited by a CCL8 inhibitor. These results indicate that at least some of ZEB1′s pro-tumour functions require and are mediated mainly by CCL8 in cancer cells and CCR2 in macrophages. Targeting tumour-infiltrating macrophages via CCL8–CCR2 signalling showed promising potential for TAM-based strategies for cervical cancer treatment.

The NF-κB signalling pathway is important in cancer-related inflammation and malignant progression^44,45^. Recently, its important role in maintaining the M2 phenotype and pro-tumoural function in TAMs has been highlighted in various cancers^44,45^. Our results showed that CCL8 increased phosphorylated p65 expression through CCR2. Inhibition of NF-κB by PDTC reversed CCL8-induced macrophage migration, suggesting that CCL8 mediates the activation of the NF-κB pathway. This is consistent with previous findings in oesophageal squamous cancer cells^38^. Quinones et al.^46^ also reported that the expression of CCR2 leads to the activation of NF-κB. Additional data showed that hypoxia-sensitive NF-κB signalling is critical for the activation of chemotactic genes such as CCL2, CCL3, and VEGF^47^, which will potentiate TAM infiltration. The NF-κB pathway is also of key importance in the transcriptional regulation of immunity, and it controls multiple aspects of immune cell function. Activated NF-κB signalling is essential for maintaining the immunosuppressive phenotype of TAMs^48^. Our data, together with previous studies, indicate that NF-κB may serve as a downstream signal of the CCL8–CCR2 axis, suggesting that the NF-κB pathway is the molecular signal responsible for ZEB1-mediated TAM infiltration.

Once macrophages are attracted to the hypoxic TME, their mobility is slowed down via hypoxia-dependent mechanisms^49^. Macrophages are primed to serve pro-tumoural functions when entrapped in the hypoxic niche. Our previous study verified that the cervical hypoxic TME induced the recruited macrophages to transform into a pro-tumoural M2 phenotype^7^. Moreover, the increase in PD-L1 and IDO in hypoxic macrophages subsequently exhausted the T cells, which aggravated therapeutic resistance^50,51^. Interestingly, TAMs in the hypoxic TME could also release CCL8 and thus cooperate with cancer cells to attract macrophages into the hypoxic area and promote tumour progression^8^. As an important inducer driving EMT, hypoxia-induced ZEB1 modulated the interaction between cancer cells and TAMs, and this crosstalk, in turn, regulated ZEB1 expression itself.

In summary, this study shows that hypoxia-induced ZEB1 potentiates TAM infiltration through the promotion of CCL8 to form a metastasis-favourable TME; thus, we uncovered a novel role for the ZEB1–CCL8 axis as a key factor in the infiltration of TAMs and propose that targeting the communication between hypoxic cancer cells and TAMs could be a promising strategy for the prevention of cervical cancer progression.

## Supplementary information

Figure S1

Figure S2

Supplementary figure legends
