# Correction: Hypoxia-induced ZEB1 promotes cervical cancer progression via CCL8-dependent tumour-associated macrophage recruitment

**DOI:** 10.1038/s41419-022-04706-y

**Published:** 2022-03-17

**Authors:** Xiao-Jing Chen, Yuan-Run Deng, Zi-Ci Wang, Wen-Fei Wei, Chen-Fei Zhou, Yan-Mei Zhang, Rui-Ming Yan, Luo-Jiao Liang, Mei Zhong, Li Liang, Sha Wu, Wei Wang

**Affiliations:** 1 Department of Obstetrics and Gynecology, Nanfang Hospital, Southern Medical University, 510515 Guangzhou, China; 2 Department of Obstetrics and Gynecology, The First Affiliated Hospital of Guangzhou Medical University, 510120 Guangzhou, China; 3 Department of Obstetrics and Gynecology, The Third Affiliated Hospital, Southern Medical University, 510360 Guangzhou, China; 4 Department of Immunology, School of Basic Medical Sciences, Southern Medical University, Guangdong Provincial Key Laboratory of Proteomic, 510515 Guangzhou, China; 5 Department of Pathology, Nanfang Hospital, Southern Medical University, 510515 Guangzhou, China; 6 Present Address: 1838 Guangzhou Avenue North, Baiyun District, 510515 Guangzhou, China; 7 Present Address: 151 Yanjiang Road, Yuexiu District, 510120 Guangzhou, China

**Keywords:** Cancer microenvironment, Cervical cancer, Checkpoint signalling

Correction to: *Cell Death & Disease* 10.1038/s41419-019-1748-1, published online 1 July 2019

The authors would like to correct Figs. 2 and 4, as errors were introduced in the preparation of these figures for publication. The authors declare that these corrections do not change the results or conclusions of this paper. We sincerely apologize for having these errors in the article, and apologize for any inconvenience caused. The authors have provided corrected version of Figs. 2 and 4 here.

The revised Fig. 2 is as follows:

**Fig. 2:**
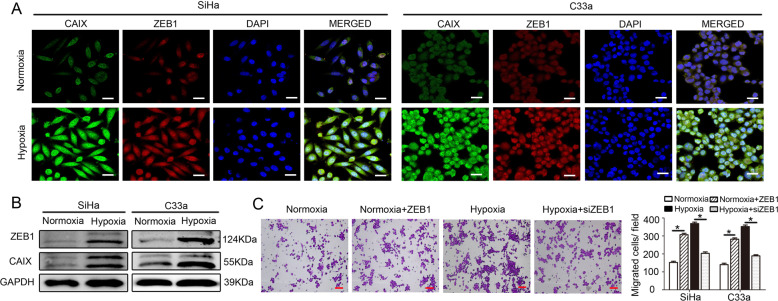
Hypoxia-induced ZEB1 in cancer cells enhances TAM migration in vitro. **a** Immunofluorescence revealed that hypoxic SiHa and C33a cells exhibited upregulated expression of CAIX (green) and ZEB1 (red). Nuclei were stained with DAPI (blue) (scale bar, 50 μm). **b** Western blotting shows that the protein levels of both ZEB1 (upper panel) and CAIX (middle panel) were higher in hypoxic cancer cells than in normoxic cancer cells (left panel shows SiHa cells; right panel shows C33a cells). **c** The effect of conditioned media (CM) from hypoxia-treated cervical cancer cells on THP-1 cells was detected by transwell migration assay. Scale bar, 50 μm. *P < 0.05 by Student’s *t*-test.

The revised Fig. 4 is as follows:

**Fig. 4:**
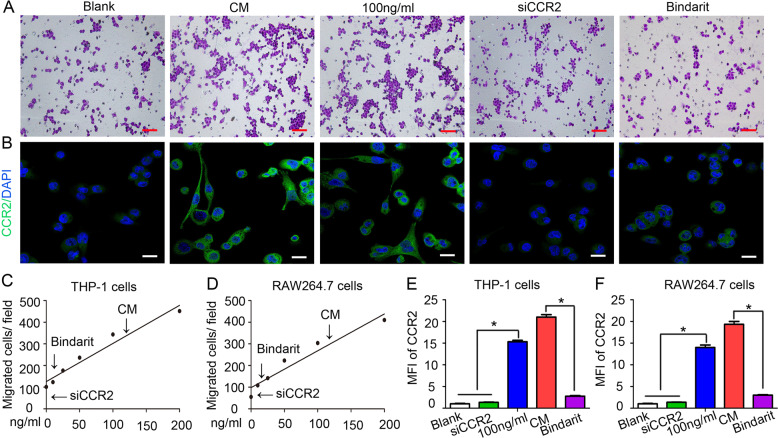
CCL8-CCR2 interaction mediates TAM infiltration under hypoxic conditions. **a** Using a migration test, we identified that the migration effect of hypoxic cervical cancer cells on THP-1 cells was almost equivalent to that of 100 ng/ml CCL8. Bindarit (a CCL8 synthesis inhibitor) and siCCR2 significantly impaired the directional migration of THP-1 cells. Scale bar, 100 μm. **b** Immunofluorescence staining revealed that the level of CCR2 expression was increased in THP-1 cells incubated with 100 ng/ml CCL8 and decreased in THP-1 cells treated with bindarit or siCCR2. Scale bar, 100 μm. **c** Statistical analysis showing the number of migrated THP-1 cells. **d** Statistical analysis showing the number of migrated RAW264.7 cells. **e** Statistical analysis showing the fold change of the mean fluorescence intensity (MFI) of CCR2 in THP-1 cells. **f** Statistical analysis showing the fold change of the MFI of CCR2 in RAW264.7 cells.

