# Supplementary figures and images for: Hypoxia-induced ZEB1 promotes cervical cancer progression via CCL8-dependent tumour-associated macrophage recruitment
Source: Cell Death Dis. 2019 Jul 1;10(7):508. doi: 10.1038/s41419-019-1748-1 (PMC6602971; doi:10.1038/s41419-019-1748-1)

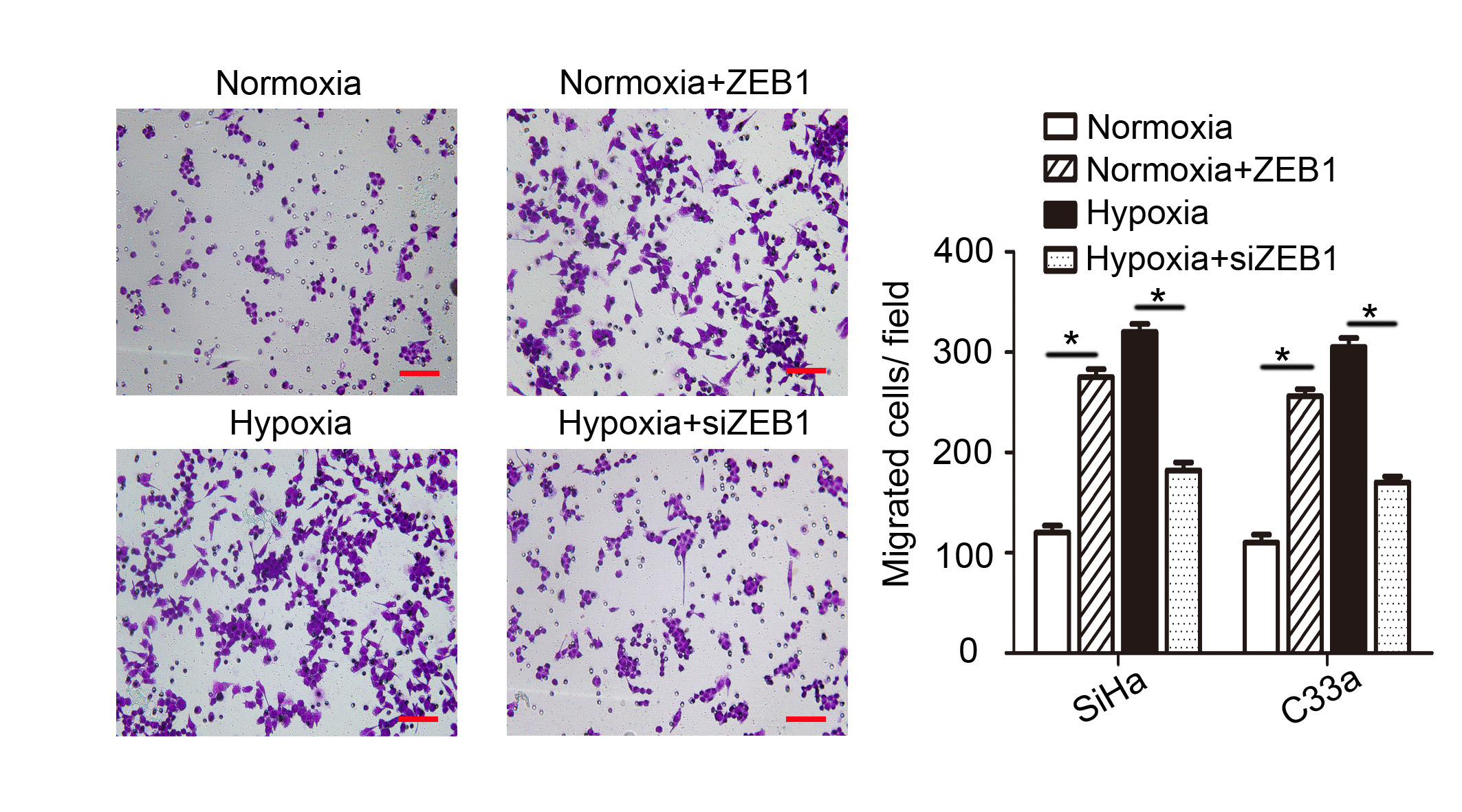

Supplement: Supplementary file 1 — Figure S1 [file 41419_2019_1748_MOESM1_ESM.jpg]

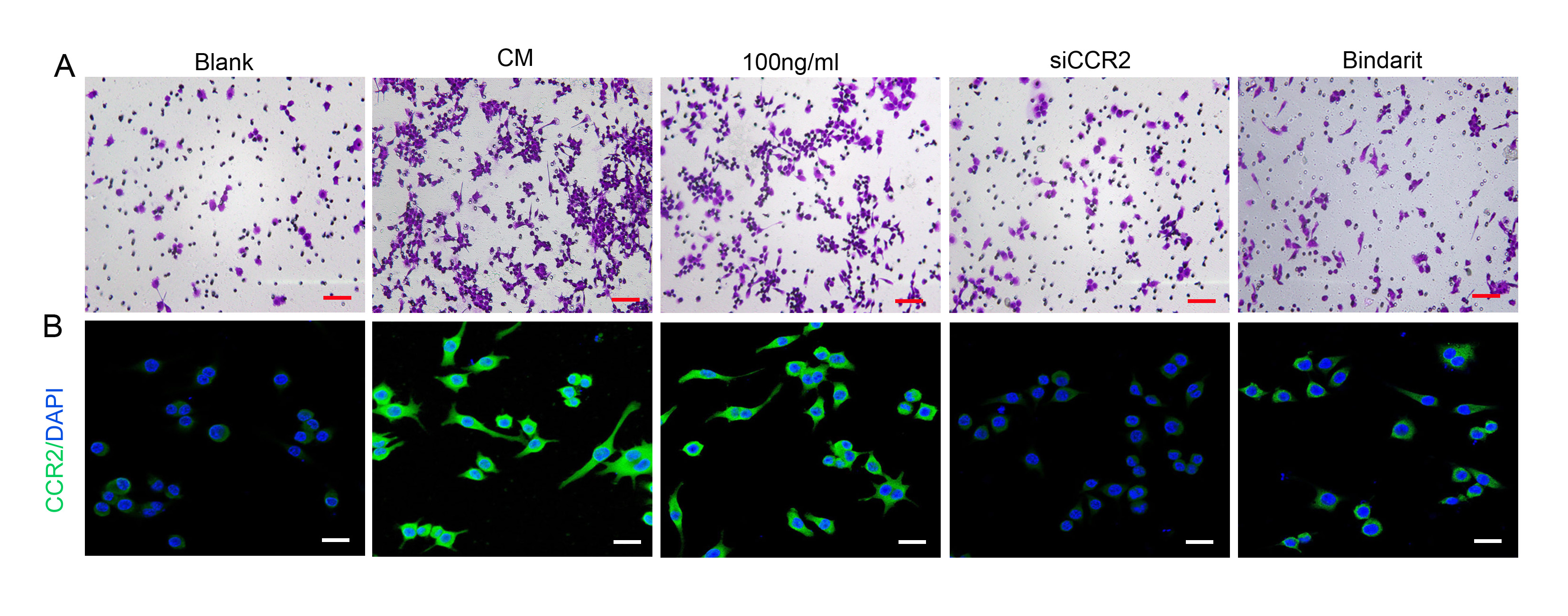

Supplement: Supplementary file 2 — Figure S2 [file 41419_2019_1748_MOESM2_ESM.jpg]
