# Supplementary material for: Hypoxia-induced ZEB1 promotes cervical cancer progression via CCL8-dependent tumour-associated macrophage recruitment
Source: Cell Death Dis. 2019 Jul 1;10(7):508. doi: 10.1038/s41419-019-1748-1 (PMC6602971; doi:10.1038/s41419-019-1748-1)
Supplement: Supplementary file 3 — Supplementary figure legends [file 41419_2019_1748_MOESM3_ESM.docx]

**Supplementary Figure Legends**

**Figure S1.** The number of RAW264.7 cells migrating towards the CM of hypoxic cancer cells was increased significantly compared to that migrating towards the CM of normoxic cancer cells. ZEB1 overexpression in normoxic cervical cancer cells significantly increased the migration of RAW264.7 cells, whereas ZEB1 depletion in hypoxic cervical cancer cells had the opposite effect. Scale bar, 50 μm. **P*<0.05 by Student’s t-test.

**Figure S2.** The CCL8-CCR2 interaction is involved in TAM migration. (A) Using a migration test, we identified that the migration effect of hypoxic cervical cancer cells on RAW264.7 cells was almost equivalent to that of 100 ng/ml CCL8. Bindarit and siCCR2 significantly impaired the directional migration of RAW264.7 cells. Scale bar, 100 μm. (B) Immunofluorescence staining revealed that the level of CCR2 expression was increased in RAW264.7 cells incubated with 100 ng/ml CCL8 and decreased in RAW264.7 cells treated with bindarit or siCCR2. Scale bar, 100 μm.
